# Identification of therapeutic targets and prognostic biomarkers from the hnRNP family in invasive breast carcinoma

**DOI:** 10.18632/aging.202411

**Published:** 2021-01-20

**Authors:** Jiawei Zhou, Yugang Guo, Zheng Huo, Yuxin Xing, Jintao Fang, Guohui Ma, Qinghui Han, Mengzhen Wang, Qian Xu

**Affiliations:** 1 Henan Provincial Engineering Laboratory of Insects Bio-Reactor, Nanyang Normal University, NanYang 473000, China; 2 School of Chemistry and Pharmaceutical Engineering, Nanyang Normal University, NanYang 473000, China

**Keywords:** heterogeneous nuclear ribonucleoproteins, invasive breast carcinoma, metastasis, prognostic markers

## Abstract

Heterogeneous nuclear ribonucleoproteins (hnRNPs) are RNA-binding proteins that are reported to play a crucial role in the pathogenic process of multiple malignancies. However, their expression patterns, clinical application significance and prognostic values in invasive breast carcinoma (BRCA) remain unknown. In this study, we investigated hnRNP family members in BRCA using accumulated data from Oncomine 4.5, UALCAN Web portal and other available databases. We explored the expression and prognostic value level of hnRNPs in BRCA. We further analyzed their association with the clinicopathological features of BRCA patients. Subsequently, we calculated the alteration frequency of hnRNPs, constructed the interaction network of hnRNPs, and examined the potential coexpression genes of hnRNPs, revealing that HNRNPU and SYNCRIP are the core molecular genes requiring further investigation for BRCA. We validated the immunohistochemistry (IHC) pattern to simulate clinical applications based on pathology. Cell function experiments conducted *in vitro* indicated that HNRNPU can promote epithelial-mesenchymal transition, functionally stimulating the invasion capacity and inhibiting the viability of invasive BRCA cells. In summary, our systematic analysis demonstrated that HNRNPU was the key molecule that played a fundamental role in BRCA metastasis, which may facilitate the development of new diagnostic and prognostic markers for the analysis of BRCA progression.

## INTRODUCTION

Breast carcinoma (BRCA) is the leading cause of cancer-associated mortality in women worldwide [1]. The pathogenesis of BRCA is extremely complex, reflecting the diversity in clinical subcategories at the cellular level [1, 2]. The most common subtype of BRCA is invasive ductal carcinoma (IDC), accounting for approximately 60%–75% of all BRCA cases. On the other hand, invasive lobular carcinoma (ILC) accounts for 5%–15% of all BRCA cases and is the second most common type of invasive BRCA [3]. Despite recent advances in BRCA therapy, tumor metastasis remains the leading cause of mortality in patients with BRCA. Therefore, it is imperative to identify prognostic markers and potential drug targets as well as understand the mechanisms underlying BRCA development in order to improve prognosis and promote individualized treatment.

Heterogeneous nuclear ribonucleoproteins (hnRNPs) represent a large family of RNA-binding proteins with various key cellular functions. The hnRNP family members are attracting increasing attention with respect to their association with cancer occurrence and progression [4, 5]. Cellular functions are dysregulated during tumorigenesis, including alternative splicing and translational and RNA processing [5–8]. Accumulating experimental evidence indicates that the hnRNP family members play pivotal roles in multiple cancers. For example, the increased expression of HNRNPA2B1 leads to tumor suppression in multiple cancers, including pancreatic ductal adenocarcinoma, ovarian cancer, hepatocellular carcinoma, lung cancer, melanoma, glioblastoma, and prostate cancer [9–15]. HNRNPC is negatively associated with the overall survival of patients with advanced gastric cancer treated with 5FU-based drugs [16]. HNRNPLL has been reported to modulate the alternative splicing of CD44 during epithelial–mesenchymal transition (EMT) in colorectal cancer metastasis [17]. HNRNPM is frequently associated with high-grade human breast tumor and promotes BRCA metastasis [18]. However, the different expression levels and the specific functions of individual hnRNPs members in BRCA, and association between hnRNPs and the clinicopathological features of BRCA remain unelucidated [19]. In addition, how and when hnRNPs are activated to interact and functionally complement each other remain unknown. Therefore, an in-depth investigation and analysis into the potential roles of hnRNPs in BRCA are warranted.

In the present study, we attempted to explore and analyze the expression patterns and clinical significance of hnRNPs in BRCA using integrated bioinformatics. In addition, we sought to determine whether hnRNPs can predict the progression and prognosis of patients with BRCA. We hope that this study will serve as a meaningful reference for understanding the roles of hnRNPs in BRCA as well as for improving the effectiveness of treatment and prognostic accuracy of patients with BRCA.

## RESULTS

### Expression levels of the hnRNP family members in patients with BRCA

**Figure 1:**
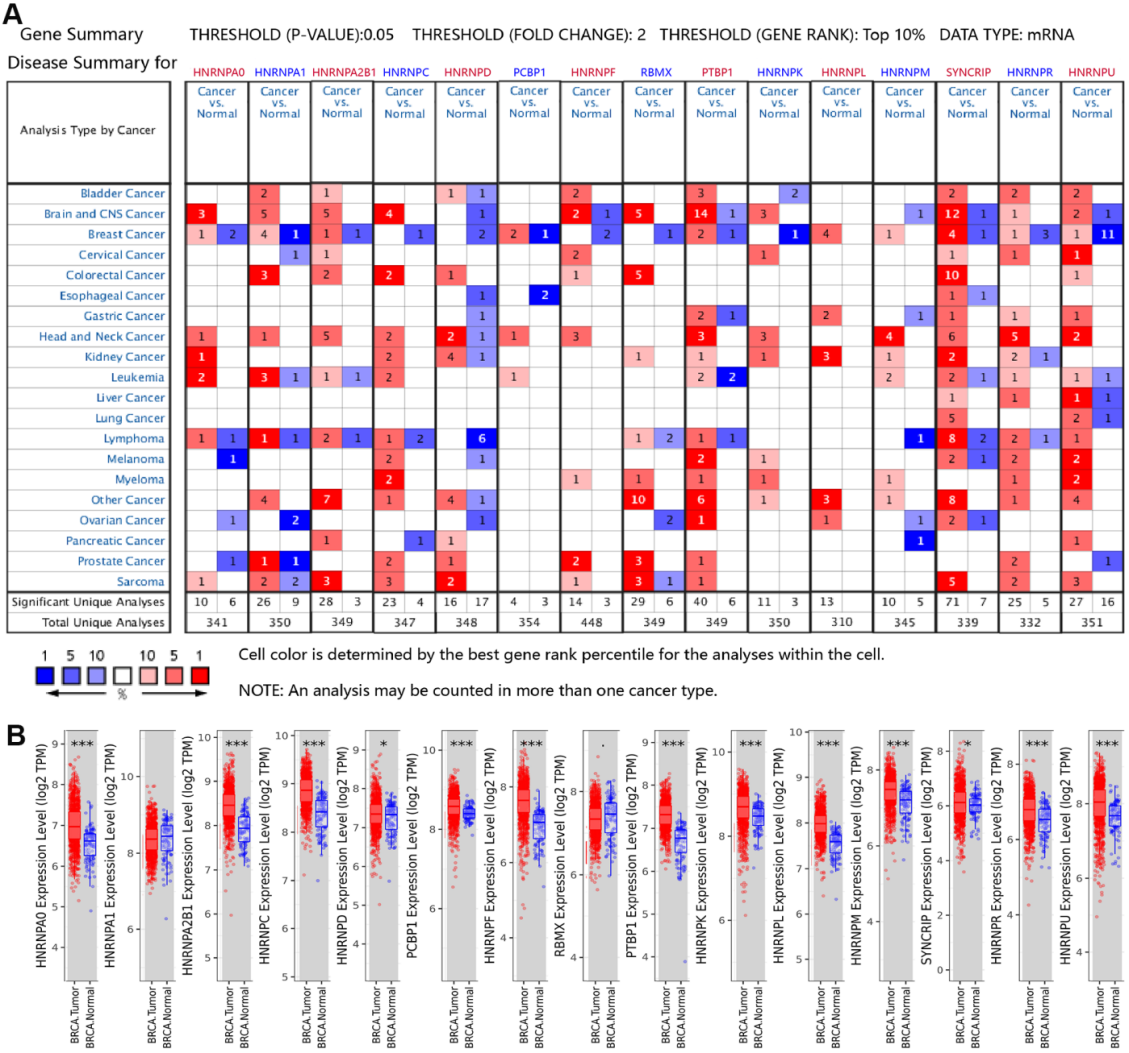
**Aberrant mRNA expression of hnRNPs in BRCA.** (**A**) mRNA expression of hnRNPs in various cancer types based on the Oncomine database. The Figure shows the number of datasets with statistically significant mRNA overexpression (red) or downregulated expression (blue) of hnRNPs in different types of cancer versus normal tissues. The threshold was designed with a P value of 0.05, fold change of 2, and gene ranking of 10%. The gene-rank percentile was analyzed for the top target gene from all genes measured in each research. Cell color was determined by the best gene-rank percentile for analysis within the cell. (**B**) Box plots showing the distribution of the expression of the hnRNPs across BRCA and normal tissue samples in the TCGA-BRCA dataset.*P < 0.05; **P < 0.01; ***P < 0.001.

The mRNA expression patterns of the hnRNPs were determined using the Oncomine and The Cancer Genome Atlas (TCGA) databases. Oncomine analysis revealed that there were significant changes in hnRNP expression at the transcriptional level between different types of cancer and normal tissues following the threshold search criteria (*P* value < 0.05; fold change > 2, and top 10% gene rank). The significant unique analyses between cancer and normal tissues that met our selection criteria in pan-cancer analysis are shown in Figure 1A and Supplementary Table 1. HNRNPA0, HNRNPA1, HNRNPA2B1, PCBP1, HNRNPL, HNRNPM, SYNCRIP, and HNRNPU were significantly upregulated in the two main subtypes of BRCA (IDC and ILC) compared with in normal tissues in the datasets by Gluck et al. [20], Radvanyi et al. [21], Ma et al. [22] and Turashvili et al. [23]. As shown in Figure 1B, in the TCGA cohort, HNRNPA0, HNRNPA2B1, HNRNPC, HNRNPD, PCBP1, HNRNPF, HNRNPK, HNRNPL, HNRNPM, PTBP1, SYNCRIP, HNRNPR, and HNRNPU were found to be upregulated in 1,093 invasive BRCA tissue specimens compared with in 112 normal tissue specimens from the TCGA cohort (*P* < 0.05). The results of Radvanyi et al. showed that the mRNA expression levels of HNRNPA1, HNRNPL, and HNRNPR were notably increased in invasive mixed BRCA tissues than in normal tissues. Radvanyi et al. and Ma et al. revealed that HNRNPA, PCBP1, HNRNPL, and SYNCRIP were upregulated in ductal BRCA tissues in situ than in normal tissues. However, Finak et al. [24] reported significantly lower expression levels of HNRNPA0, HNRNPA1, HNRNPA2B1, HNRNPC, HNRNPD, PCBP1, HNRNPF, RBMX, HNRNPK, and HNRNPU in BRCA-associated stroma than in normal tissues. The results of Curtis from the European Genome-phenome Archive demonstrated that HNRNPU was downregulated in invasive BRCA tissues than in normal tissues. These findings suggest that the expression of hnRNP family members is associated with patients in different areas or different histological types of BRCA.

### Correlation of hnRNP mRNA expression with individual stages of cancer, lymph node metastases, and pathological subtypes in TCGA-BRCA

**Figure 2:**
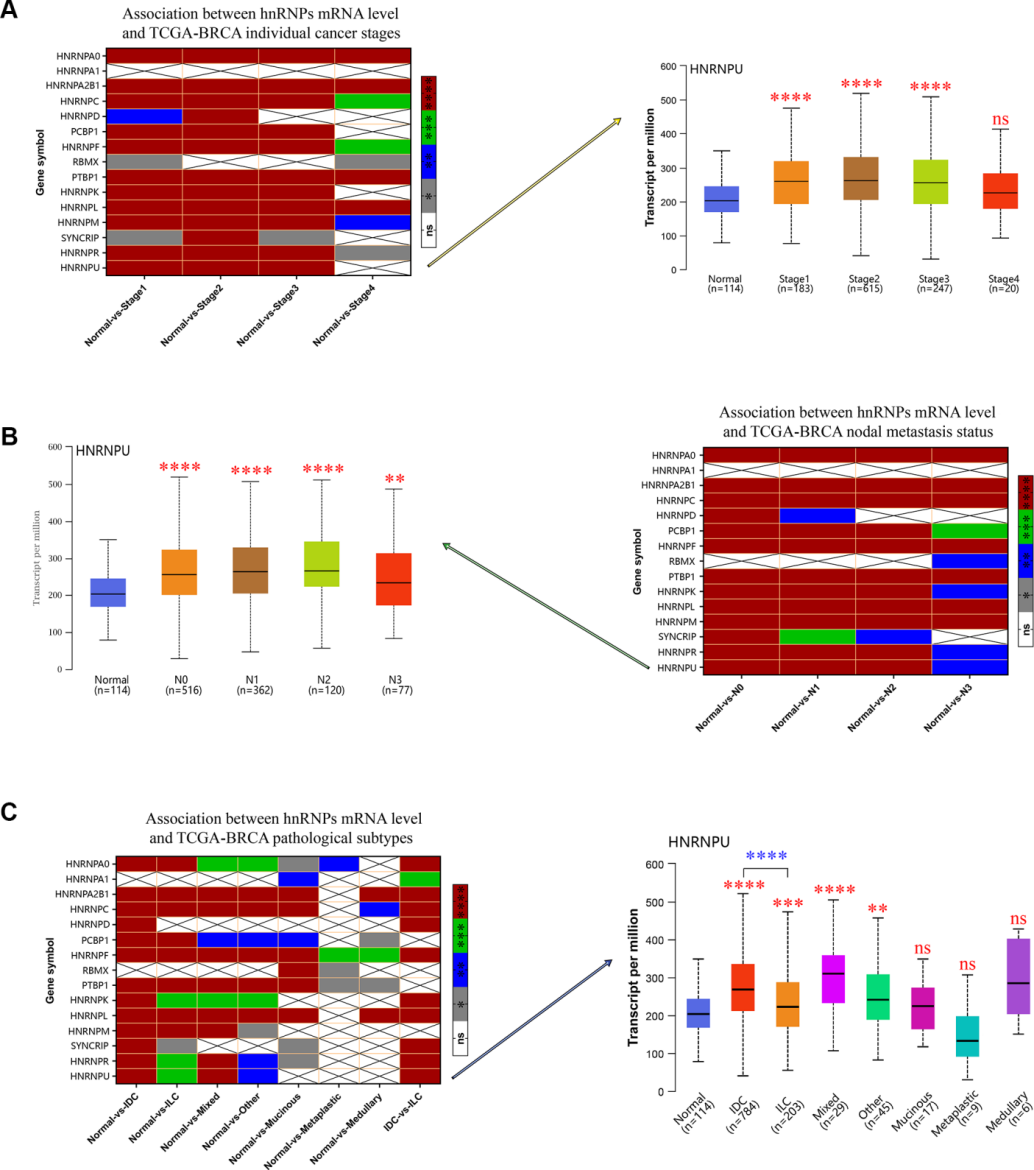
**The relationship between the mRNA expression of hnRNPs and tumor stage, nodal metastasis status, and pathological subtypes of patients with TCGA-BRCA.** (**A**) Heat map displaying the correlation between the expression of hnRNPs and tumor stage. The boxplot shows the correlation between HNRNPU expression and tumor stages. (**B**) Heat map displaying the association between the expression of hnRNPs and nodal metastasis status. The boxplot shows the association between HNRNPU expression and the nodal metastasis status. (**C**) Heat map displaying the correlation between the expression of hnRNPs and tumor pathological subtypes. The boxplot shows the association between hnRNPU expression and tumor pathological subtypes. Gray represents *P < 0.05, blue represents **P < 0.01, green represents ***P < 0.001, red represents ****P < 0.0001, and white represents no significant difference.

We evaluated the relationship between the mRNA expression levels of hnRNPs and the different clinicopathological subgroups of patients in the TCGA-BRCA cohort. As shown in Figure 2, significant differences between the groups were assessed to generate *P* values and then illustrated using heat maps. These data indicated that the upregulated mRNA expression levels of HNRNPA0, HNRNPA2B1, HNRNPC, HNRNPD, PCBP1, HNRNPF, PTBP1, HNRNPK, HNRNPL, HNRNPM, SYNCRIP, HNRNPR, and HNRNPU were significantly associated with high cancer stages and lymph node metastases (LNM) with few exceptions. However, the mRNA expression level of HNRNPA1 was not associated with cancer stages and LNM in patients with BRCA (*P* > 0.05; Figure 2A, 2B). Except for HNRNPA1 and RBMX, the expression levels of the remaining members were higher in IDC tissues than in normal tissues (*P* < 0.0001; Figure 2C). In addition, except for HNRNPA1, HNRNPD, and RBMX, the expression levels of the remaining 12 members were higher in ILC tissues than in normal tissues (*P* < 0.05; Figure 2C). These results suggest that the mRNA expression levels of HNRNPA0, HNRNPA1, HNRNPA2B1, HNRNPC, HNRNPD, HNRNPF, HNRNPK, HNRNPL, SYNCRIP, HNRNPR, and HNRNPU are markedly higher in IDC tissues than in ILC tissues (*P*< 0.001; Figure 2C), indicating that hnRNPs play significant roles in the tumorigenesis and progression of BRCA, particularly IDC and ILC. However, owing to the limited sample size of some histological groups (mixed, other, mucinous, medullary, or metaplastic), we did not present an in-depth analysis of their subgroup comparison data.

### Prognostic value of hnRNPs in BRCA

**Figure 3:**
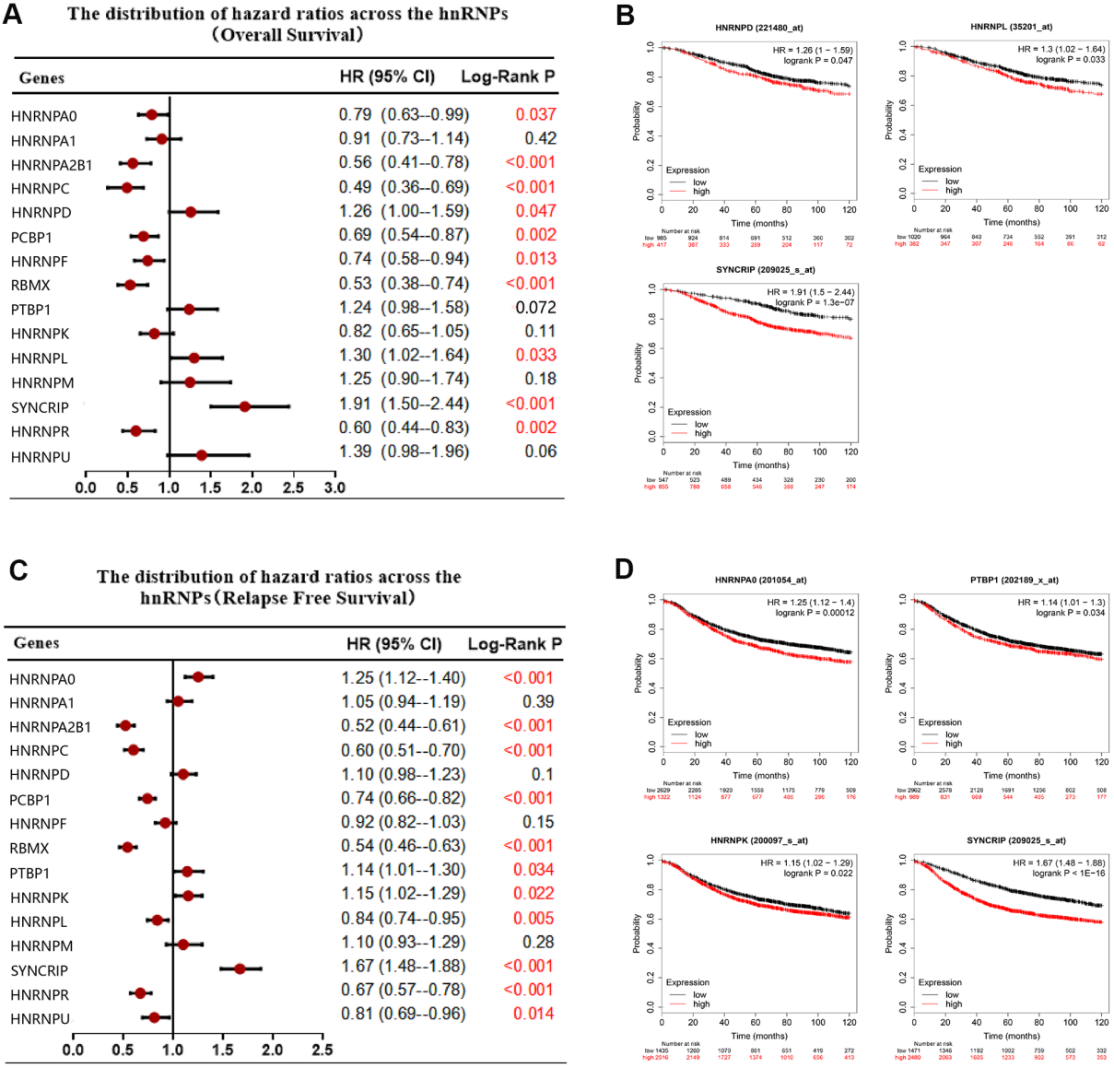
**Correlation between hnRNP mRNA expression and clinical prognosis in patients with BRCA.** (**A**) Forest plots showing the correlation between the expression of hnRNPs and patient OS, as examined via Kaplan–Meier analysis. (**B**) High hnRNPD, hnRNPL, or SYNCRIP expression was significantly associated with poor OS. (**C**) Forest plots showing the association between the mRNA expression levels of hnRNPs and RFS. (**D**) High expression of HNRNPA0, PTBP1, HNRNPK, and SYNCRIP was significantly associated with poor RFS.

To date, the potential prognostic value of only few members of the hnRNP family have been investigated in certain cohorts. Therefore, to analyze the independent prognostic value of the hnRNP family members, we analyzed the prognostic role of 15 hnRNP family members in patients with BRCA using the Kaplan–Meier (KM) method. As shown in Figure 3A, 3B, 10 of the 15 tested genes significantly correlated with OS (*P* < 0.05). Among these genes, HNRNPD, HNRNPL, and SYNCRIP were risky genes, with a hazard ratio (HR) of >1 and log-rank *P* value of <0.05. In contrast, HNRNPA0, HNRNPA2B1, HNRNPC, PCBP1, HNRNPF, RBMX, and HNRNPR were protective genes, with an HR of <1 and log-rank *P* value of <0.05. In addition, the mRNA expression levels of HNRNPA0, HNRNPA2B1, HNRNPC, PCBP1, RBMX, PTBP1, HNRNPK, HNRNPL, SYNCRIP, HNRNPR, and HNRNPU were associated with recurrence-free survival (RFS) in patients with BRCA (*P* < 0.05; Figure 3C). In particular, among these 11 genes, increased mRNA expression levels of HNRNPA0, PTBP1, HNRNPK, and SYNCRIP were associated with poor RFS in patients with BRCA under the selected best cutoff value (*P* < 0.05; Figure 3D).

Despite recent advances in BRCA therapy, metastasis remains the leading cause of mortality in patients with BRCA. Subgroup analysis revealed that the increased expression of HNRNPU significantly indicated a poor OS in patients with BRCA with LNM (HR, 2.11; *P* = 0.029), However, the increased expression of HNRNPA0, HNRNPC, HNRNPF, and HNRNPK predicted improved survival in patients with BRCA with LNM (HR, <1; *P* < 0.05). RFS data analysis revealed that the increased expression levels of HNRNPA0, HNRNPA2B1, HNRNPC, HNRNPD, HNRNPF, HNRNPK, HNRNPR, and HNRNPU were associated with improved RFS in patients with BRCA with LNM (HR, <1; *P* < 0.05). In contrast, the increased expression of PTBP1 (HR, 1.25; *P* = 0.032), HNRNPL (HR, 1.73; *P* <.0001), HNRNPM (HR, 1.42; *P* = 0.01), and SYNCRIP (HR, 1.84; *P* <.0001) were associated with poor RFS in patients with BRCA with LNM (Supplementary Table 2).

### Protein expression levels of hnRNPs in BRCA and their association with the clinicopathological features of patients with BRCA

**Figure 4:**
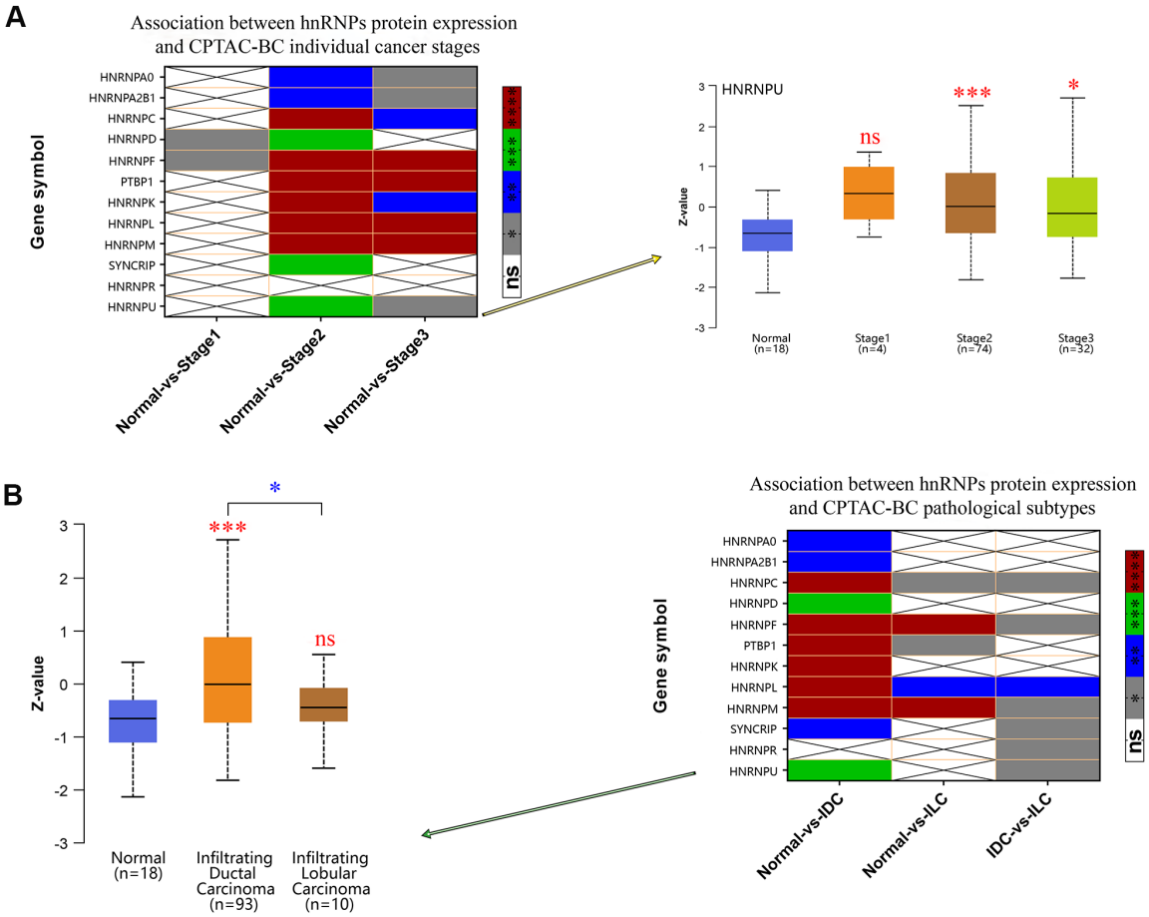
**Correlation between hnRNP protein expression, tumor stage, and pathological subtypes.** (**A**) Heat map displaying the correlation between hnRNP protein expression and tumor stage. The boxplot shows the association between HNRNPU protein expression and tumor stage. (**B**) Heat map displaying the correlation between hnRNP protein expression and tumor pathological subtypes. The boxplot shows the association between HNRNPU protein expression and tumor pathological subtypes. Gray represents *P < 0.05, blue represents **P < 0.01, green represents ***P < 0.001, red represents ****P < 0.0001, and white represents no significant difference.

Because HNRNPA1 and RBMX were not altered in 1,093 patients with invasive BRCA compared with in 112 normal tissues from the TCGA cohort, the mRNA expression level of PCBP1 is not associated with LNM in BRCA. Therefore, we further identified the protein expression levels of the other 12 genes in patients with BRCA using the Clinical Proteomic Tumor Analysis Consortium (CPTAC) database. Our results indicated that HNRNPA2B1, HNRNPC, HNRNPD, HNRNPF, PTBP1, HNRNPK, HNRNPL, HNRNPM, SYNCRIP, and HNRNPU were upregulated in BRCA tissues compared with in normal tissues (Supplementary Figure 1), which corroborated the mRNA expression results detailed in the previous section of this manuscript. We analyzed the association between the protein expression profile and clinicopathological data and found that increased protein expression levels of HNRNPA2B1, HNRNPC, HNRNPD, HNRNPF, PTBP1, HNRNPK, HNRNPL, HNRNPM, and HNRNPU were associated with high cancer stages (Figure 4A). Further, we found that the protein expression levels of HNRNPA2B1, HNRNPC, HNRNPD, HNRNPF, PTBP1, HNRNPK, HNRNPL, HNRNPM, SYNCRIP, and HNRNPU were higher in IDC tissues than in normal tissues and that the protein expression levels of HNRNPC, HNRNPF, PTBP1, HNRNPL, and HNRNPM were higher in ILC tissues than in normal tissues. In addition, the expression levels of HNRNPC, HNRNPF, HNRNPL, HNRNPM, SYNCRIP, HNRNPR, and HNRNPU were significantly higher in IDC tissues than in ILC tissues (Figure 4B; *P* < 0.05).

### Genetic mutation, interaction, and coexpression analyses of hnRNPs

**Figure 5:**
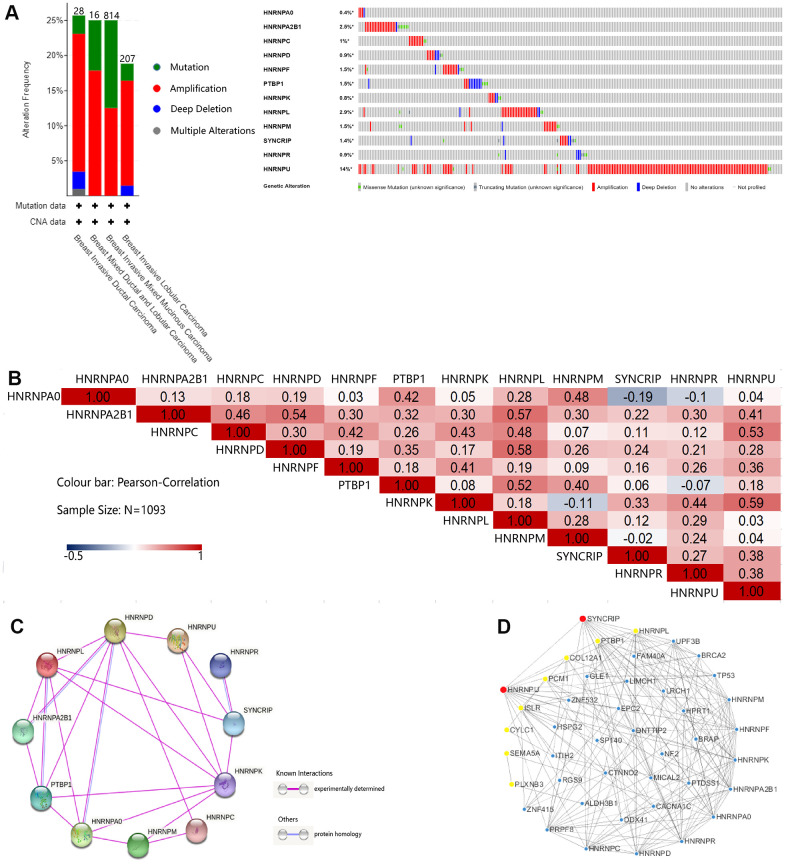
**Genetic mutation, interaction, and coexpression analyses of hnRNPs in patients with BRCA.** (**A**) Summary of the alteration frequencies of nodal metastasis-related hnRNPs in patients with TCGA-BRCA (Breast Invasive Carcinoma, TCGA). The number of patients for each BRCA subtype is indicated at the top of each column and each type of alteration, including deletions (blue), amplification (red), multiple alterations (gray), or mutations (green), is shown using the cBioPortal. (**B**) Correlation plot based on Spearman’s correlation test results to show the correlation of gene expression among the 12 hnRNP family members in BRCA. (**C**) PPI among hnRNPs was derived from STRING. The purple connection indicates the protein interaction determined by the experiment and the blue connection indicates that the proteins are homologous. (**D**) The 12 hnRNP family members and coexpressed gene networks were screened using Cytoscape. Red and yellow represent the top 10 hub genes.

The different mutation spectra seen in the geographical and ethnic populations can be used to identify the environmental exposure contributing toward BRCA development. We considered their possible roles in BRCA risk and their association with the type of cancer developed. Therefore, we calculated the alteration frequency of hnRNPs across the TCGA-BRCA cohort using the cBioPortal online tool. hnRNPs were altered in 595 samples from 1065 patients (60%) in the TCGA-BRCA cohort (Figure 5A). Notably, HNRNPU, HNRNPL, and HNRNPA2B1 were the three most frequently altered genes (14%, 2.9%, and 2.5%, respectively), and amplification, mutation, and deep deletion were the three most common types of mutations. We also confirmed the alteration frequency of hnRNPs in the two other datasets (Metastatic Breast Cancer Project Provisional and Breast Cancer; METABRIC) (Supplementary Figure 2A, 2B). Similar to the results of the TCGA-BRCA cohort, HNRNPU was the most frequently altered gene in these two datasets (13% and 23%, respectively). Next, we explored the potential coexpression genes of hnRNPs. Pearson’s correlation analysis revealed the following significant correlations: HNRNPA0 with PTBP1 and HNRNPM; HNRNPA2B1 with HNRNPC, HNRNPD, HNRNPL, and HNRNPU; HNRNPC with HNRNPF, HNRNPK, HNRNPL, and HNRNPU; HNRNPD with HNRNPL; HNRNPF with HNRNPK; PTBP1 with HNRNPL and HNRNPM; and HNRNPK with HNRNPR and HNRNPU (R > 0.4; N = 1093; Figure 5B). These results indicate that several hnRNPs interact with each other during the pathogenesis of BRCA. We conducted a protein–protein interaction (PPI) network analysis of hnRNPs to present the interaction among the 12 genes using STRING. As shown in Figure 5C, the purple connecting line shows the correlation among the genes when experimentally determined. We then constructed another network for hnRNPs and the most frequently altered coexpressed genes. The results showed that 43 genes, including BRCA2, TP53, ZNF532, CYLC1, COL12A1, SEMA5A, PLXNB3, and PCM1, were closely related with alterations in hnRNPs. Using Cytoscape, we found that of the 12 hnRNPs, HNRNPU, SYNCRIP, HNRNPL, and PTBP1 were the hub genes in the network (Figure 5D). Next, we studied the potential biological processes of the top 50 genes that positively and negatively correlated with HNRNPU mutation in BRCA using Linkomics (Supplementary Figure 2C–2E). The Gene Ontology (GO) term functional enrichment analyses of these genes are summarized (*P* < 0.01) in Supplementary Figure 2F–2H. Among the GO terms for biological processes, the top five terms were “protein binding,” “ion binding,” “molecular transducer activity,” “hydrolase activity,” and “transporter activity.”

### Immunohistochemistry (IHC)-predicted patterns for potential clinical applications

**Figure 6:**
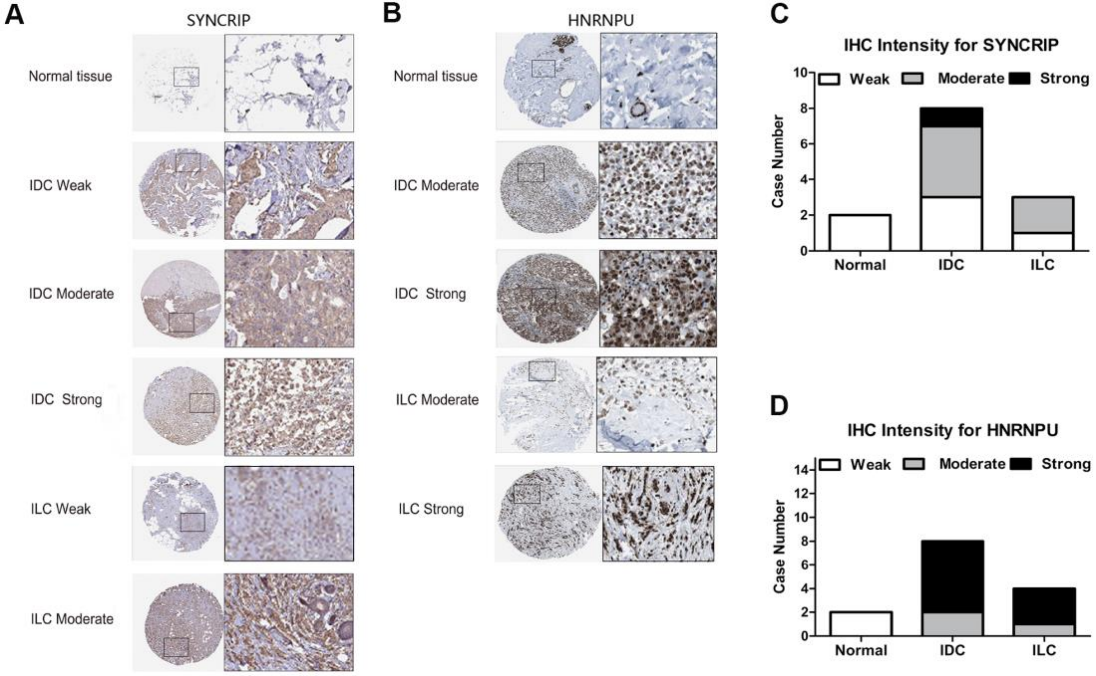
**The IHC expression pattern of SYNCRIP and HNRNPU in normal breast tissues and BRCA.** (**A**, **B**) Representative images are shown for strong, moderate, weak and negative expression of SYNCRIP and HNRNPU in normal breast tissues and BRCA (IDC and ILC). The black rectangle indicates a higher magnification of the indicated area in the image. (**C**) Bar chart of the IHC staining intensity of SYNCRIP for BRCA (a total of 13 cases). (**D**) Bar chart of the IHC staining intensity of hnRNPU for BRCA (a total of 14 cases).

Because HNRNPU and SYNCRIP were identified as the core molecular genes and SYNCRIP positively correlated with OS and HNRNPU positively correlated with tumor stage and metastasis, we further validated the IHC pattern from the Human Protein Atlas database to simulate clinical applications on the basis of pathology. Several IHC images of hnRNP proteins displayed a trend toward differential expression in BRCA subtype samples (IDC or ILC) compared with in normal tissue samples. These results were in accordance with the transcriptional level of hnRNPs. SYNCRIP staining showed negative or low staining in the cytoplasm and cell membranes of normal tissues, and HNRNPU staining showed negative staining in the cytoplasm and cell membrane. In BRCA (IDC and ILC) tissues, strong and moderate staining patterns of SYNCRIP were clearly located in the nucleus, cytoplasm, and cell membrane. For HNRNPU, moderate and strong patterns were mainly located in the nucleus, cytoplasm, and cell membrane. This unique IHC staining of BRCA could help distinguish cancer tissues from normal tissues as well as predict clinical outcomes (Figure 6A, 6B). The relative staining intensity of SYNCRIP was negative (two cases) for normal tissues; weak (three cases), moderate (four cases), and strong (one case) for IDC tissues; and weak (one case) and moderate (two cases) for ILC tissues (Figure 6C). For HNRNPU staining, both cases were weak for normal tissue; moderate (two cases) and strong (six cases) for IDC tissues; and moderate (one case) and strong (three cases) for ILC tissues (Figure 6D).

### HNRNPU promoted breast cancer cell proliferation, migration, and invasion

**Figure 7:**
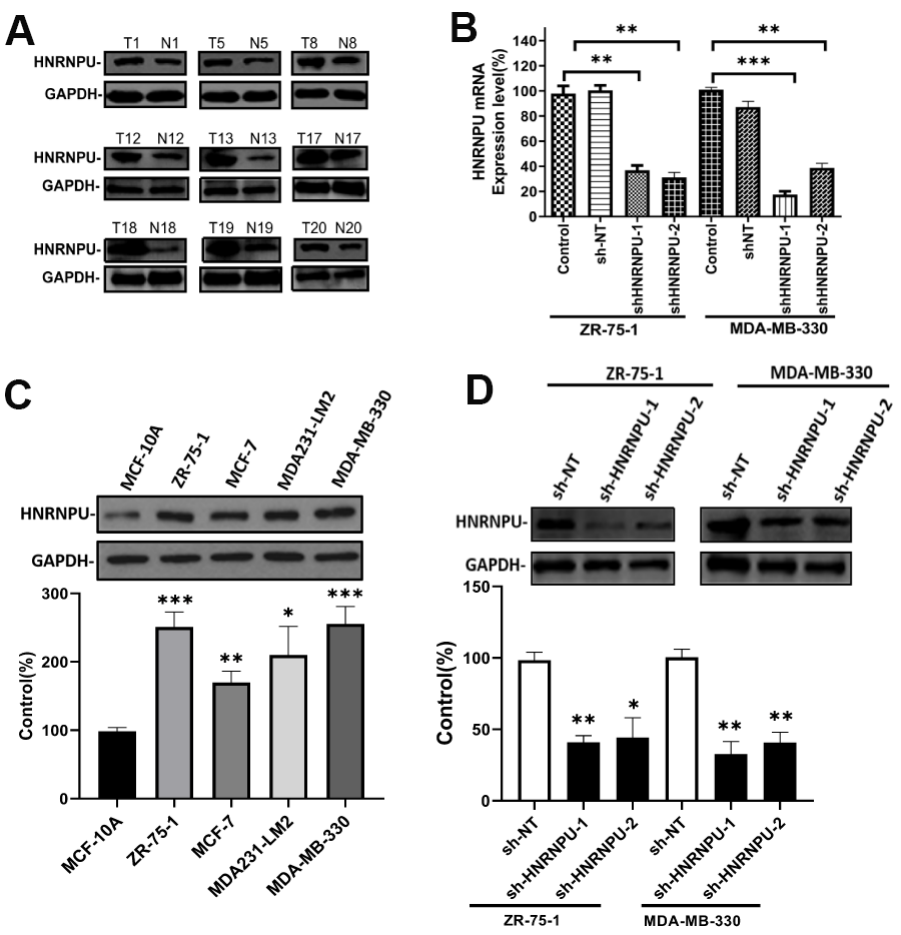
**HNRNPU expression is upregulated in BRCA cell lines and clinical tumor samples.** Altering expression of HNRNPU proteins in clinical tumor samples (**A**) and four human BRCA cell lines (**B**), as determined via western blot analysis. Glyceraldehyde 3-phosphate dehydrogenase was used as the control. ZR-75-1 and MDA-MB-330 cells were transfected with shRNA and the mRNA (**C**), and protein (**D**) expression levels of HNRNPU were measured via reverse transcription quantitative real-time polymerase chain reaction and western blotting. *P < 0.05; **P < 0.01; ***P < 0.001.

**Figure 8:**
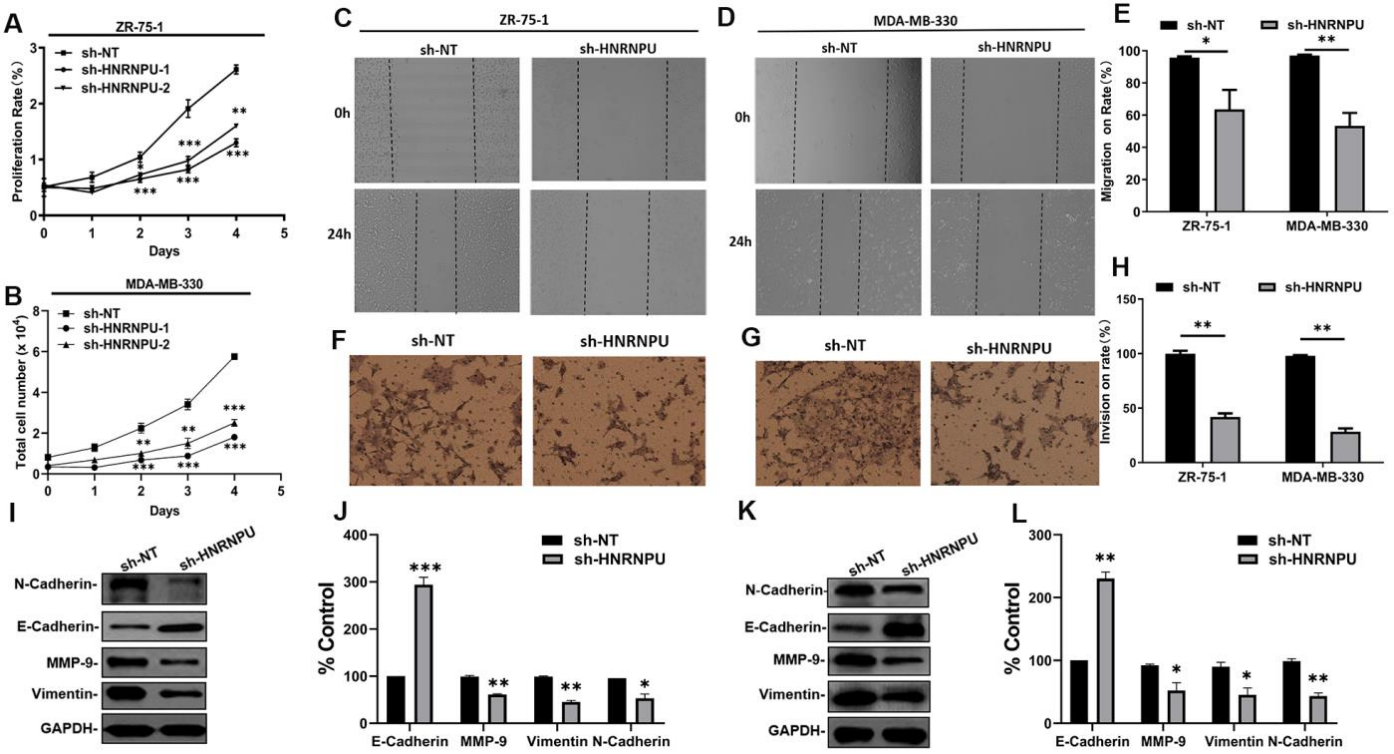
**HNRNPU improved metastatic ability of cells by activating EMT phenotypes in BRCA cells.** (**A**, **B**) Effects of HNRNPU knockdown on the proliferation of ZR-75-1 and MDA-MB-330 cells. (**C**–**E**) Representative images from the Transwell migration assays for ZR-75-1 and MDA-MB-330 cells transfected with sh-HNRNPU and sh-NT. (**F**–**H**) Representative images from the wound-healing assays for ZR-75-1 and MDA-MB-330 cells transfected with sh-HNRNPU and sh-NT. (**I**–**L**) Effects of HNRNPU knockdown on the expression of EMT signal protein detection in ZR-75-1 (**I**, **J**) and MDA-MB-330 (**K**, **L**) cells using western blotting. *P < 0.05; **P < 0.01; ***P < 0.001 compared with sh-NT.

Because HNRNPU was discovered as a key gene in BRCA among the hnRNP family members and was associated with nodal metastasis in BRCA, we speculated that HNRNPU is involved in the regulation of BRCA metastasis. To test this hypothesis, we first investigated the expression of HNRNPU in 32 breast IDC and adjacent normal tissues. The expression level of HNRNPU was higher in IDC tissues than in normal tissues (Figure 7A). We then examined the expression level of HNRNPU in a series of BRCA cell lines (MCF-7, ZR-75-1, MDA-MB-330, and MDA231-LM2) and a normal breast epithelial cell line (MCF-10A). The expression level of HNRNPU was higher in the BRCA cell lines than in MCF-10A cells (Figure 7B). To investigate the functional roles of HNRNPU in invasive BRCA progression, HNRNPU expression was knocked down using shRNAs in ZR-75-1 and MDA-MB-330 cells (Figure 7C, 7D). MTT assay revealed that HNRNPU knockdown substantially reduced the proliferation ability of ZR-75-1 and MDA-MB-330 cells (Figure 8A, 8B). Moreover, the wound-healing and Matrigel invasion assays revealed that HNRNPU knockdown significantly inhibited the migration and invasion abilities of ZR-75-1 and MDA-MB-330 cells (*P* < 0.05, Figure 8C–8H). Further analysis of HNRNPU knockdown in ZR-75-1 and MDA-MB-330 cells revealed that HNRNPU depletion can increase the expression level of the epithelial marker E-cadherin and decrease the expression levels of the mesenchymal markers vimentin, N-cadherin, and matrix metallopeptidase 9, suggesting that HNRNPM is important for the EMT phenotype (Figure 8I–8L). Taken together, our results suggest that HNRNPU knockdown significantly inhibits the proliferation, migration, and invasion of BRCA cells *in vitro*. Therefore, we reached the tentative conclusion that HNRNPU is the key molecule that plays a fundamental role in BRCA metastasis.

## DISCUSSION

BRCA is one of the most common malignancies in women, with 1.20 million cases reported annually in women worldwide [1]. Moreover, approximately one in eight women in the United States develops BRCA during the course of her lifetime. Its incidence is expected to increase globally [25]. Although several molecular factors have emerged as predictive markers of BRCA, it remains challenging to clarify the prognostic significance of those biomarkers.

Accumulated evidence indicates that hnRNPs play a variety of potential roles in the inhibition of apoptosis, angiogenesis, cell invasion, and EMT. The significant association among the hnRNP family members and clinicopathological features and survival outcomes of patients with cancer has been confirmed, indicating that hnRNPs are novel and promising cancer therapeutic targets and predictive biomarkers for treatment response and prognostic evaluation [16, 26–31]. In BRCA, the hnRNP family members, including HNRNPA2B1, HNRNPC, HNRNPH1, HNRNPK, HNRNPM, and PTBP1, function as oncogenes in BRCA progression [7, 8, 18, 32–36]. Liu et al. reported that HNRNPA2B1 is a negative regulator of BRCA metastasis [36]. Hu et al. revealed that HNRNPA2B1 knockdown inhibits cell proliferation, induces apoptosis, and prolonges the S phase of the cell cycle in MCF-7 and MDA-MB-231 cells via the STAT3 and ERK1/2 signaling pathways [37]. Wu et al. showed that HNRNPC repression inhibits cell proliferation and tumor growth in BRCA cells [38]. Yang et al. reported that HNRNPM promotes BRCA progression by regulating the axin/β-catenin signaling pathway [34]. In addition, HNRNPH1 has been reported to result in the production of HER2 splice variants in BRCA [7]. PTBP1 has been shown to promote the growth of BRCA cells via the PTEN/Akt pathway and autophagy [8]. As there are few studies on other members of the hnRNP family, understanding the initial molecular events leading to BRCA development may provide opportunities for prophylactic intervention, improve our ability to predict the risk of BRCA, and offer advanced strategies for early detection. Therefore, in our study, we provided the first systemic analysis of the hnRNP family members in BRCA as therapeutic targets for the diagnosis, prognosis, and treatment of BRCA.

The results of our study showed that the mRNA expression levels of HNRNPA0, HNRNPA2B1, HNRNPC, HNRNPD, PCBP1, HNRNPF, HNRNPK, HNRNPL, HNRNPM, PTBP1, SYNCRIP, HNRNPR, and HNRNPU were upregulated in patients with BRCA, whereas those of HNRNPA1 and RBMX were not altered in BRCA tissues compared with in normal tissues based on the TCGA dataset. Clinical correlation analysis revealed that increased mRNA and protein expression levels of HNRNPA2B1, HNRNPC, PCBP1, HNRNPF, HNRNPK, HNRNPL, HNRNPM, SYNCPIP, and HNRNPU were observed in subgroups with high pathological stage and LNM. To validate the prognostic indicators, we found that increased mRNA expression levels of HNRNPD, HNRNPL, and SYNCRIP were associated with poor OS rates and that increased mRNA expression levels of HNRNPA0, PCBP1, HNRNPK, and SYNCRIP were associated with poor RFS rates. Furthermore, subgroup analysis revealed that high expression of HNRNPU significantly indicated poor OS in patients with BRCA with LNM. In addition, we found that increased expression levels of HNRNPA0, HNRNPA2B1, HNRNPC, HNRNPD, HNRNPF, PTBP1, HNRNPK, HNRNPL, HNRNPM, SYNCRIP, and HNRNPU proteins positively associated with tumor stages. Moreover, the unique IHC staining of SYNCRIP and HNRNPU from BRCA could distinguish cancer tissues from normal tissues and help clinicians predict the clinical outcome. Therefore, the expression of hnRNPs (SYNCRIP and HNRNPU) in BRCA may be further validated clinically as a potential diagnostic and prognostic marker.

The progression of the invasion and migration of cancer cells is propelled by different functions and interaction of multiple genes at multiple steps. In recent years, EMT has been believed to be a key step in the invasion and metastasis of cancer cells [39]. Previous studies have shown that several hnRNP family members are implicated in the regulation of EMT in several human cancer types and are regarded as cancer metastasis suppressors [17, 18, 20, 40, 41]. We investigated the function of HNRNPU in regulating EMT. In our study, we first detected the expression of HNRNPU in 32 patients with BRCA cancer and found that its expression was considerably higher in cancer tissues than in adjacent tissues. In addition, we found that HNRNPU suppressed human BRCA metastasis by promoting EMT development, functionally stimulating the invasion ability and inhibiting the viability of invasive BRCA cell lines. These results suggest that HNRNPU could be used as a potential biomarker to predict the prognosis of patients with IDC.

Accumulating evidence suggests that RNA epigenetics has received increased attention in the past few years [4]. The hnRNP family members not only play an important role in alternative splicing but also participate in the regulation of microRNAs (miRNAs) or long non-coding RNAs [4, 42]. Studies have reported that HNRNPA2/B1 binds to m6A markers in a part of the miRNA transcripts and promotes primary miRNA processing. Half of the miRNAs regulated by m6A depend on HNRNPA2/B1 [42, 43]. Vascular endothelial growth factor A transcript expression in hypoxic conditions is regulated by HNRNPL competing with miRNA binding in the 3′-untranslated region of vascular endothelial growth factor A [44]. Similarly, PTBP1 binds to let-7 miRNA and human Argonaute 2, thereby altering their association with human mRNAs [45]. Many hnRNPs appear in the same complex, indicating that multiple hnRNPs have the same structure and function. Studies on the regulation of alternative splicing in cells may begin with the direction of the interaction mechanism between the low-complexity regions of hnRNPs and m6A-modified RNA as development progresses.

In conclusion, in this study, we aimed to understand the clinical significance of the hnRNP family in BRCA and the molecular mechanism based on big data. We hope that our findings offer new perspectives in future research and clinical applications for patients with BRCA. However, some potential limitations of this study should be discussed. Although we confirmed that hnRNPs play crucial roles in the pathogenesis of BRCA, additional experiments are warranted to confirm the association between hnRNPs and BRCA as new indicators in large patient cohorts.

## MATERIALS AND METHODS

### Ethics statement

The study has been conducted in accordance with the ethical standards and the Declaration of Helsinki as well as according to national and international guidelines. All procedures were approved by the Ethics Committee of the Institute of Clinical Pharmacology. Informed consent has been obtained.

### Bioinformatics analysis

During the validation process, the transcription levels of hnRNP in different types of cancer were analyzed using the Oncomine 4.5 (https://www.oncomine.org) gene expression array dataset [46] and TIMER (https://cistrome.shinyapps.io/timer/) [47]. The mutation frequency of hnRNPs in a variety of cancers was analyzed based on TCGA, and a detailed diagram was constructed for hnRNPs in BRCA samples based on mutations. CNA data were analyzed using the online database cBioPortal (https://www.cbioportal.org/) [48]. To intuitively analyze the relationship between the mRNA and protein expression levels of hnRNPs and clinicopathology, we compared the patterns of the expression levels of hnRNPs in major BRCA types and downloaded a boxplot based on these patterns from the UALCAN web portal (http://ualcan.path.uab.edu/), which is an interactive Web portal that provides gene expression analyses based on TCGA and MET500 cohort data as well as protein expression analysis based on CPTAC data [49]. The comparison between hnRNPs expression and TCGA-BRCA tumor pathological subtypes, lymph metastasis status, and major subclasses were selected to create heat maps. Additionally, LinkedOmics (http://www.linkes.org/login.php) was used to analyze the mRNA expression levels of hnRNP-coexpression genes in the TCGA-BRCA cohort based on Pearson’s correlation coefficient [50]. All results were graphically presented in a heat map. To analyze OS and RFS, we divided the patients into two groups based on the best-performing threshold using KM plotter (https://www.kmplot.com). Next, the distribution of HR with 95% confidence intervals of OS and RFS across the hnRNPs was selected to create forest plots. Further, to examine the differences in the protein expression levels of hnRNPs between IDC, ILC, and normal tissues, we obtained the immunohistochemistry data of SYNCRIP and HNRNPU from the HPA database (https://www.proteinatlas.org/) [51].

### Patient samples

All samples (IDC and adjacent normal tissues) were obtained from Nanyang Second People's Hospital (Nanyang, Henan, China) between October 2019 and June 2020 (n = 32). All pathological features were confirmed by experienced pathologists, and none of the patients received preoperative anticancer treatment.

### Cell culture

The human breast cell lines MCF-10A, ZR-75-1, MCF-7, MDA231-LM2, and MDA-MB-330 were obtained from the Type Culture Collection of the Chinese Academy of Sciences (Shanghai, China). Cells were cultured in Dulbecco’s modified Eagle medium (Invitrogen, Shanghai, China) supplemented with 10% fetal bovine serum (Gibco, Logan, UT) and 100 U of penicillin and streptomycin at 37° C in a humidified atmosphere containing 5% CO2.

### Cell transfection

The shRNA for the specific inhibition of HNRNPU expression and a negative control shRNA were synthesized by GenePharma Co., Ltd. (Shanghai, China). Exponentially growing untreated cells were plated 24 h before transfection. The plated cells were transfected with HNRNPU shRNA using 5 μL of Lipo-RNAiMAX following the manufacturer’s instructions (Invitrogen, Carlsbad, CA, USA). After treatment, the cells were processed for further analysis.

### RNA extraction and quantitative real-time polymerase chain reaction

Total RNA was extracted from the cultured cells or tissue samples using the TRIzol reagent (Invitrogen) following the manufacturer’s instructions. First-strand cDNA synthesis was performed using the Prime Script RT Master Mix (TaKaRa, Dalian, China) and reverse transcription quantitative real-time polymerase chain reaction was performed using the SYBR Green PCR kit (TaKaRa) according to the manufacturer’s instructions. Glyceraldehyde 3-phosphate dehydrogenase was used as an endogenous control. The gene expression was calculated using the 2−ΔΔCt method. All data represent the average of three replicates.

### Cell viability assays

One day after transfection, cells were re-seeded into 96-well plates. Cell viability was measured using a Cell Counting Kit 8 assay (Dojindo Laboratories, Kumamoto, Japan), according to the manufacturer's instructions. The absorbance value of each well was measured at 450 nm. Five replicates were set for each group and the whole experiment was repeated three times.

### Wound-healing assay

Cells were seeded in 6-well plates and were transfected with the shRNA after 24 h. After culturing the cells for an appropriate amount of time, artificial wounds were made using a micropipette tip. The wounded monolayers were washed with phosphate-buffered saline (PBS) to remove cell debris. The distance between the two edges of the wound was calculated and imaged at 0 and 24 h under a microscope.

### Transwell invasion assays

For the invasion assays, cells were seeded in a 24-well Corning FluoroBlok chamber precoated with Matrigel (BD Biosciences, Billerica, MA, USA). A medium containing 20% FBS was added to the lower chamber in a 5% CO_2_ incubator at 37° C for 24 h. Then, the cells remaining on the lower side of the membrane were stained with 4% paraformaldehyde for 15 min, stained with 0.1% cresyl violet, washed three times with PBS, and air-dried. Five fields were randomly selected for counting the number of migrated cells and images (200× magnification) were captured under a phase-contrast microscope.

### Western blot analysis

Standard Western blotting protocols were followed [18]. Whole-cell lysates were probed with antibodies specific to GAPDH (Santa Cruz, CA, USA), HNRNPU, MMP9, E-Cadherin, Vimentin, and N- Cadherin (Cell Signaling, UK).

### Statistical analysis

Data are expressed as the mean ± standard error of the mean for at least three independent experiments. Analysis of variance was used to distinguish the differences in each group. *P* < 0.05 was considered statistically significant. All analyses were performed using GraphPad Prism version 8.0 (Graphpad Software, CA, USA).

## Supplementary Material

Supplementary Figures

Supplementary Table 1

Supplementary Table 2

## References

[1] Chen W. Cancer statistics: updated cancer burden in China. Chin J Cancer Res. 2015; 27:1. 10.3978/j.issn.1000-9604.2015.02.07 25717219 PMC4329178

[2] Auweter SD, Allain FH. Structure-function relationships of the polypyrimidine tract binding protein. Cell Mol Life Sci. 2008; 65:516–27. 10.1007/s00018-007-7378-2 17975705 PMC11131600

[3] Bandyopadhyay S, Bluth MH, Ali-Fehmi R. Breast carcinoma: updates in molecular profiling 2018. Clin Lab Med. 2018; 38:401–20. 10.1016/j.cll.2018.02.006 29776638

[4] Wu B, Su S, Patil DP, Liu H, Gan J, Jaffrey SR, Ma J. Molecular basis for the specific and multivariant recognitions of RNA substrates by human hnRNP A2/B1. Nat Commun. 2018; 9:420. 10.1038/s41467-017-02770-z 29379020 PMC5789076

[5] Meredith EK, Balas MM, Sindy K, Haislop K, Johnson AM. An RNA matchmaker protein regulates the activity of the long noncoding RNA HOTAIR. RNA. 2016; 22:995–1010. 10.1261/rna.055830.115 27146324 PMC4911922

[6] Geuens T, Bouhy D, Timmerman V. The hnRNP family: insights into their role in health and disease. Hum Genet. 2016; 135:851–67. 10.1007/s00439-016-1683-5 27215579 PMC4947485

[7] Gautrey H, Jackson C, Dittrich AL, Browell D, Lennard T, Tyson-Capper A. SRSF3 and hnRNP H1 regulate a splicing hotspot of HER2 in breast cancer cells. RNA Biol. 2015; 12:1139–51. 10.1080/15476286.2015.1076610 26367347 PMC4829299

[8] Wang X, Li Y, Fan Y, Yu X, Mao X, Jin F. PTBP1 promotes the growth of breast cancer cells through the PTEN/Akt pathway and autophagy. J Cell Physiol. 2018; 233:8930–39. 10.1002/jcp.26823 29856478 PMC6175200

[9] Barceló C, Etchin J, Mansour MR, Sanda T, Ginesta MM, Sanchez-Arévalo Lobo VJ, Real FX, Capellà G, Estanyol JM, Jaumot M, Look AT, Agell N. Ribonucleoprotein HNRNPA2B1 interacts with and regulates oncogenic KRAS in pancreatic ductal adenocarcinoma cells. Gastroenterology. 2014; 147:882–92.e8. 10.1053/j.gastro.2014.06.041 24998203

[10] Yang Y, Wei Q, Tang Y, Wang Y, Luo Q, Zhao H, He M, Wang H, Zeng Q, Lu W, Xu J, Liu T, Yi P. Loss of hnRNPA2B1 inhibits Malignant capability and promotes apoptosis via down-regulating Lin28B expression in ovarian cancer. Cancer Lett. 2020; 475:43–52. 10.1016/j.canlet.2020.01.029 32006618

[11] Chettouh H, Fartoux L, Aoudjehane L, Wendum D, Clapéron A, Chrétien Y, Rey C, Scatton O, Soubrane O, Conti F, Praz F, Housset C, Rosmorduc O, Desbois-Mouthon C. Mitogenic insulin receptor-A is overexpressed in human hepatocellular carcinoma due to EGFR-mediated dysregulation of RNA splicing factors. Cancer Res. 2013; 73:3974–86. 10.1158/0008-5472.CAN-12-3824 23633480

[12] Dai L, Li J, Tsay JJ, Yie TA, Munger JS, Pass H, Rom WN, Tan EM, Zhang JY. Identification of autoantibodies to ECH1 and HNRNPA2B1 as potential biomarkers in the early detection of lung cancer. Oncoimmunology. 2017; 6:e1310359. 10.1080/2162402X.2017.1310359 28638733 PMC5467997

[13] Li T, Gu M, Deng A, Qian C. Increased expression of YTHDF1 and HNRNPA2B1 as potent biomarkers for melanoma: a systematic analysis. Cancer Cell Int. 2020; 20:239. 10.1186/s12935-020-01309-5 32549786 PMC7294677

[14] Golan-Gerstl R, Cohen M, Shilo A, Suh SS, Bakàcs A, Coppola L, Karni R. Splicing factor hnRNP A2/B1 regulates tumor suppressor gene splicing and is an oncogenic driver in glioblastoma. Cancer Res. 2011; 71:4464–72. 10.1158/0008-5472.CAN-10-4410 21586613

[15] Stockley J, Villasevil ME, Nixon C, Ahmad I, Leung HY, Rajan P. The RNA-binding protein hnRNPA2 regulates β-catenin protein expression and is overexpressed in prostate cancer. RNA Biol. 2014; 11:755–65. 10.4161/rna.28800 24823909 PMC4156506

[16] Huang H, Han Y, Zhang C, Wu J, Feng J, Qu L, Shou C. HNRNPC as a candidate biomarker for chemoresistance in gastric cancer. Tumour Biol. 2016; 37:3527–34. 10.1007/s13277-015-4144-1 26453116

[17] Sakuma K, Sasaki E, Kimura K, Komori K, Shimizu Y, Yatabe Y, Aoki M. HNRNPLL, a newly identified colorectal cancer metastasis suppressor, modulates alternative splicing of CD44 during epithelial-mesenchymal transition. Gut. 2018; 67:1103–11. 10.1136/gutjnl-2016-312927 28360095

[18] Xu Y, Gao XD, Lee JH, Huang H, Tan H, Ahn J, Reinke LM, Peter ME, Feng Y, Gius D, Siziopikou KP, Peng J, Xiao X, Cheng C. Cell type-restricted activity of hnRNPM promotes breast cancer metastasis via regulating alternative splicing. Genes Dev. 2014; 28:1191–203. 10.1101/gad.241968.114 24840202 PMC4052765

[19] Han SP, Tang YH, Smith R. Functional diversity of the hnRNPs: past, present and perspectives. Biochem J. 2010; 430:379–92. 10.1042/BJ20100396 20795951

[20] Glück S, Ross JS, Royce M, McKenna EF Jr, Perou CM, Avisar E, Wu L. TP53 genomics predict higher clinical and pathologic tumor response in operable early-stage breast cancer treated with docetaxel-capecitabine ± trastuzumab. Breast Cancer Res Treat. 2012; 132:781–91. 10.1007/s10549-011-1412-7 21373875

[21] Radvanyi L, Singh-Sandhu D, Gallichan S, Lovitt C, Pedyczak A, Mallo G, Gish K, Kwok K, Hanna W, Zubovits J, Armes J, Venter D, Hakimi J, et al. The gene associated with trichorhinophalangeal syndrome in humans is overexpressed in breast cancer. Proc Natl Acad Sci USA. 2005; 102:11005–10. 10.1073/pnas.0500904102 16043716 PMC1182410

[22] Ma XJ, Dahiya S, Richardson E, Erlander M, Sgroi DC. Gene expression profiling of the tumor microenvironment during breast cancer progression. Breast Cancer Res. 2009; 11:R7. 10.1186/bcr2222 19187537 PMC2687710

[23] Turashvili G, Bouchal J, Baumforth K, Wei W, Dziechciarkova M, Ehrmann J, Klein J, Fridman E, Skarda J, Srovnal J, Hajduch M, Murray P, Kolar Z. Novel markers for differentiation of lobular and ductal invasive breast carcinomas by laser microdissection and microarray analysis. BMC Cancer. 2007; 7:55. 10.1186/1471-2407-7-55 17389037 PMC1852112

[24] Finak G, Bertos N, Pepin F, Sadekova S, Souleimanova M, Zhao H, Chen H, Omeroglu G, Meterissian S, Omeroglu A, Hallett M, Park M. Stromal gene expression predicts clinical outcome in breast cancer. Nat Med. 2008; 14:518–27. 10.1038/nm1764 18438415

[25] Li CI, Anderson BO, Daling JR, Moe RE. Trends in incidence rates of invasive lobular and ductal breast carcinoma. JAMA. 2003; 289:1421–24. 10.1001/jama.289.11.1421 12636465

[26] Barboro P, Salvi S, Rubagotti A, Boccardo S, Spina B, Truini M, Carmignani G, Introini C, Ferrari N, Boccardo F, Balbi C. Prostate cancer: prognostic significance of the association of heterogeneous nuclear ribonucleoprotein K and androgen receptor expression. Int J Oncol. 2014; 44:1589–98. 10.3892/ijo.2014.2345 24626777

[27] Qiao L, Xie N, Bai Y, Li Y, Shi Y, Wang J, Liu N. Identification of upregulated HNRNPs associated with poor prognosis in pancreatic cancer. Biomed Res Int. 2019; 2019:5134050. 10.1155/2019/5134050 31355266 PMC6637714

[28] Zhou JM, Jiang H, Yuan T, Zhou GX, Li XB, Wen KM. High hnRNP AB expression is associated with poor prognosis in patients with colorectal cancer. Oncol Lett. 2019; 18:6459–68. 10.3892/ol.2019.11034 31819776 PMC6896405

[29] Xu L, Shen J, Jia J, Jia R. Inclusion of hnRNP L alternative exon 7 is associated with good prognosis and inhibited by oncogene SRSF3 in head and neck squamous cell carcinoma. Biomed Res Int. 2019; 2019:9612425. 10.1155/2019/9612425 31828152 PMC6885243

[30] Li L, Yan S, Zhang H, Zhang M, Huang G, Chen M. Interaction of hnRNP K with MAP 1B-LC1 promotes TGF-β1-mediated epithelial to mesenchymal transition in lung cancer cells. BMC Cancer. 2019; 19:894. 10.1186/s12885-019-6119-x 31492158 PMC6731588

[31] Wu S, Sato M, Endo C, Sakurada A, Dong B, Aikawa H, Chen Y, Okada Y, Matsumura Y, Sueoka E, Kondo T. hnRNP B1 protein may be a possible prognostic factor in squamous cell carcinoma of the lung. Lung Cancer. 2003; 41:179–86. 10.1016/s0169-5002(03)00226-5 12871781

[32] Mandal M, Vadlamudi R, Nguyen D, Wang RA, Costa L, Bagheri-Yarmand R, Mendelsohn J, Kumar R. Growth factors regulate heterogeneous nuclear ribonucleoprotein K expression and function. J Biol Chem. 2001; 276:9699–704. 10.1074/jbc.M008514200 11121407

[33] Zhou J, Allred DC, Avis I, Martínez A, Vos MD, Smith L, Treston AM, Mulshine JL. Differential expression of the early lung cancer detection marker, heterogeneous nuclear ribonucleoprotein-A2/B1 (hnRNP-A2/B1) in normal breast and neoplastic breast cancer. Breast Cancer Res Treat. 2001; 66:217–24. 10.1023/a:1010631915831 11510693

[34] Yang WH, Ding MJ, Cui GZ, Yang M, Dai DL. Heterogeneous nuclear ribonucleoprotein M promotes the progression of breast cancer by regulating the axin/β-catenin signaling pathway. Biomed Pharmacother. 2018; 105:848–55. 10.1016/j.biopha.2018.05.014 30021377

[35] Anantha RW, Alcivar AL, Ma J, Cai H, Simhadri S, Ule J, König J, Xia B. Requirement of heterogeneous nuclear ribonucleoprotein C for BRCA gene expression and homologous recombination. PLoS One. 2013; 8:e61368. 10.1371/journal.pone.0061368 23585894 PMC3621867

[36] Liu Y, Li H, Liu F, Gao LB, Han R, Chen C, Ding X, Li S, Lu K, Yang L, Tian HM, Chen BB, Li X, et al. Heterogeneous nuclear ribonucleoprotein A2/B1 is a negative regulator of human breast cancer metastasis by maintaining the balance of multiple genes and pathways. EBioMedicine. 2020; 51:102583. 10.1016/j.ebiom.2019.11.044 31901866 PMC6948170

[37] Hu Y, Sun Z, Deng J, Hu B, Yan W, Wei H, Jiang J. Splicing factor hnRNPA2B1 contributes to tumorigenic potential of breast cancer cells through STAT3 and ERK1/2 signaling pathway. Tumour Biol. 2017; 39:1010428317694318. 10.1177/1010428317694318 28351333

[38] Wu Y, Zhao W, Liu Y, Tan X, Li X, Zou Q, Xiao Z, Xu H, Wang Y, Yang X. Function of HNRNPC in breast cancer cells by controlling the dsRNA-induced interferon response. EMBO J. 2018; 37:e99017. 10.15252/embj.201899017 30158112 PMC6276880

[39] Thiery JP. Epithelial-mesenchymal transitions in tumour progression. Nat Rev Cancer. 2002; 2:442–54. 10.1038/nrc822 12189386

[40] Dai S, Zhang J, Huang S, Lou B, Fang B, Ye T, Huang X, Chen B, Zhou M. HNRNPA2B1 regulates the epithelial-mesenchymal transition in pancreatic cancer cells through the ERK/snail signalling pathway. Cancer Cell Int. 2017; 17:12. 10.1186/s12935-016-0368-4 28077929 PMC5223355

[41] Huang GZ, Wu QQ, Zheng ZN, Shao TR, Chen YC, Zeng WS, Lv XZ. M6A-related bioinformatics analysis reveals that HNRNPC facilitates progression of OSCC via EMT. Aging (Albany NY). 2020; 12:11667–84. 10.18632/aging.103333 32526707 PMC7343469

[42] Alarcón CR, Goodarzi H, Lee H, Liu X, Tavazoie S, Tavazoie SF. HNRNPA2B1 is a mediator of m(6)A-dependent nuclear RNA processing events. Cell. 2015; 162:1299–308. 10.1016/j.cell.2015.08.011 26321680 PMC4673968

[43] Villarroya-Beltri C, Gutiérrez-Vázquez C, Sánchez-Cabo F, Pérez-Hernández D, Vázquez J, Martin-Cofreces N, Martinez-Herrera DJ, Pascual-Montano A, Mittelbrunn M, Sánchez-Madrid F. Sumoylated hnRNPA2B1 controls the sorting of miRNAs into exosomes through binding to specific motifs. Nat Commun. 2013; 4:2980. 10.1038/ncomms3980 24356509 PMC3905700

[44] Jafarifar F, Yao P, Eswarappa SM, Fox PL. Repression of VEGFA by CA-rich element-binding microRNAs is modulated by hnRNP L. EMBO J. 2011; 30:1324–34. 10.1038/emboj.2011.38 21343907 PMC3094116

[45] Engels B, Jannot G, Remenyi J, Simard MJ, Hutvagner G. Polypyrimidine tract binding protein (hnRNP I) is possibly a conserved modulator of miRNA-mediated gene regulation. PLoS One. 2012; 7:e33144. 10.1371/journal.pone.0033144 22427970 PMC3302860

[46] Rhodes DR, Kalyana-Sundaram S, Mahavisno V, Varambally R, Yu J, Briggs BB, Barrette TR, Anstet MJ, Kincead-Beal C, Kulkarni P, Varambally S, Ghosh D, Chinnaiyan AM. Oncomine 3.0: genes, pathways, and networks in a collection of 18,000 cancer gene expression profiles. Neoplasia. 2007; 9:166–80. 10.1593/neo.07112 17356713 PMC1813932

[47] Li T, Fu J, Zeng Z, Cohen D, Li J, Chen Q, Li B, Liu XS. TIMER2.0 for analysis of tumor-infiltrating immune cells. Nucleic Acids Res. 2020; 48:W509–14. 10.1093/nar/gkaa407 32442275 PMC7319575

[48] Gao J, Aksoy BA, Dogrusoz U, Dresdner G, Gross B, Sumer SO, Sun Y, Jacobsen A, Sinha R, Larsson E, Cerami E, Sander C, Schultz N. Integrative analysis of complex cancer genomics and clinical profiles using the cBioPortal. Sci Signal. 2013; 6:pl1. 10.1126/scisignal.2004088 23550210 PMC4160307

[49] Chandrashekar DS, Bashel B, Balasubramanya SA, Creighton CJ, Ponce-Rodriguez I, Chakravarthi BV, Varambally S. UALCAN: a portal for facilitating tumor subgroup gene expression and survival analyses. Neoplasia. 2017; 19:649–58. 10.1016/j.neo.2017.05.002 28732212 PMC5516091

[50] Vasaikar SV, Straub P, Wang J, Zhang B. LinkedOmics: analyzing multi-omics data within and across 32 cancer types. Nucleic Acids Res. 2018; 46:D956–63. 10.1093/nar/gkx1090 29136207 PMC5753188

[51] Uhlén M, Fagerberg L, Hallström BM, Lindskog C, Oksvold P, Mardinoglu A, Sivertsson Å, Kampf C, Sjöstedt E, Asplund A, Olsson I, Edlund K, Lundberg E, et al. Tissue-based map of the human proteome. Science. 2015; 347:1260419. 10.1126/science.1260419 25613900

