# Supplementary material for: Identification of therapeutic targets and prognostic biomarkers from the hnRNP family in invasive breast carcinoma
Source: Aging (Albany NY). 2021 Jan 20;13(3):4503–21. doi: 10.18632/aging.202411 (PMC7906176; doi:10.18632/aging.202411)
Supplement: Supplementary Figures [file aging-13-202411-s001.pdf]

SUPPLEMENTARY FIGURES

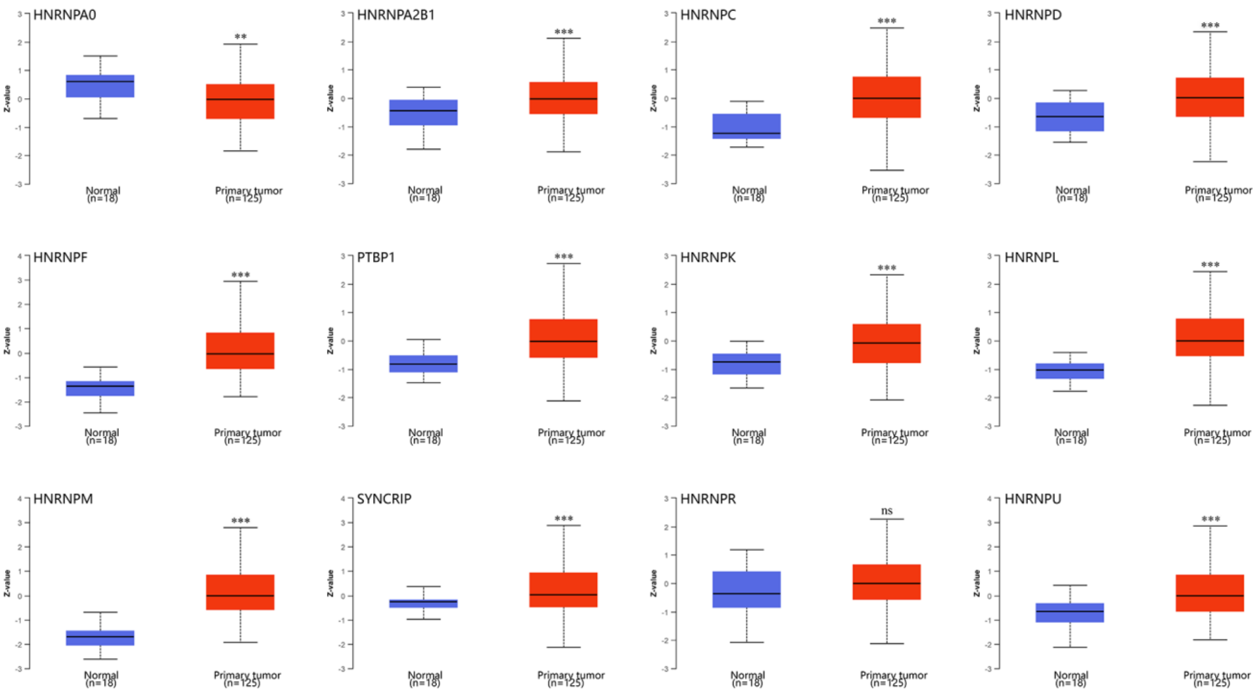

Supplementary Figure 1. Boxplot showing the relative protein expression of hnRNPs in cancer and normal tissues. Data are mean ± SE. **P* < 0.05; ***P* < 0.01; ****P* < 0.001.

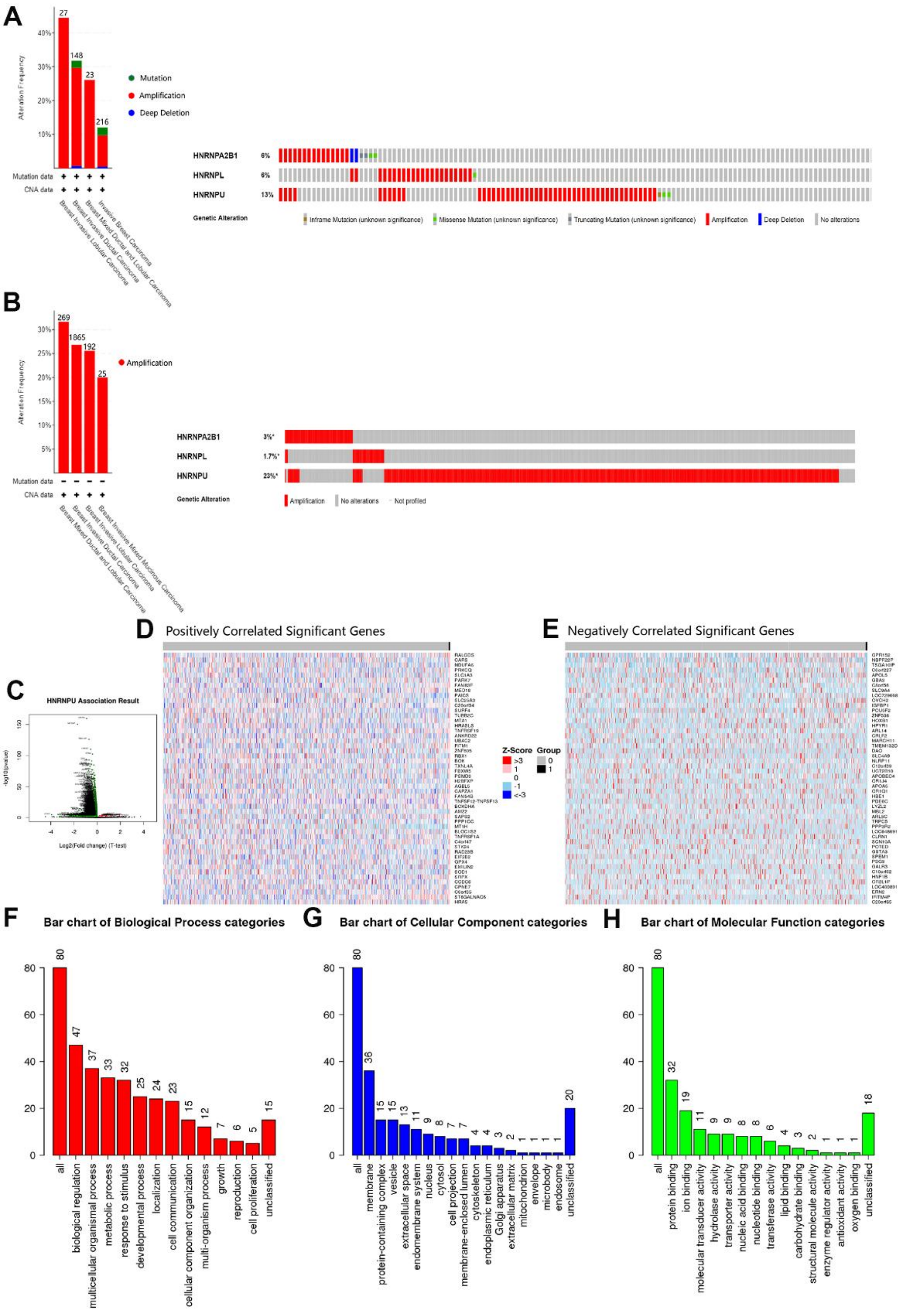

Supplementary Figure 2. Genetic mutation of HNRNPU, associated with gene expression, and enrichment analysis of differentially expressed genes in patients with TCGA-BRCA. (**A**) Verification of the top three mutations of the differentially expressed hnRNPs in patients with BRCA (The Metastatic Breast Cancer Project Provisional, February 2020, and Metastatic Breast Cancer) (INSERM, PLoS Med 2016). (**B**) Verification of the top three mutations in differentially expressed hnRNPs in BRCA (Breast Cancer METABRIC, Nature 2012 and Nat Commun 2016). (**C**) The t-test was used to analyze the effect of HNRNPU mutation on its gene expression in patients with BRCA. (**D**, **E**) Heat maps showing the genes positively and negatively correlated with HNRNPU in BRCA (top 50). The significantly enriched GO annotations of HNRNPU coexpression gene (top 100) in BRCA. (**F**) Cellular components. (**G**) Biological processes. (**H**) Molecular functions.
