# Supplementary material for: Identification of therapeutic targets and prognostic biomarkers from the hnRNP family in invasive breast carcinoma
Source: Aging (Albany NY). 2021 Jan 20;13(3):4503–21. doi: 10.18632/aging.202411 (PMC7906176; doi:10.18632/aging.202411)
Supplement: Supplementary Table 1 [file aging-13-202411-s002.docx]

**Supplementary Table 1. The mRNA levels of hnRNPs upregulated in different types of breast cancer tissues and normal tissues at transcriptome level (ONCOMINE).**

|  | **Typs of Breast Carcinoma vs. Normal** | **Fold change** | | ***P-*value** | ***t-test*** | **Ref** |
| --- | --- | --- | --- | --- | --- | --- |
|  |  | **upregulated** | **downregulated** |  |  |  |
| HNRNPA0 | Invasive Breast Carcinoma vs. Normal | 2.113 |  | <0.0001 | 8.188 | Gluck |
| HNRNPA1 | Ductal Breast Carcinoma in Situ vs. Normal | 2.565 |  | <0.05 | 2.29 | Radvanyi |
|  | Invasive Mixed Breast Carcinoma vs. Normal | 2.796 |  | <0.05 | 2.428 | Radvanyi |
|  | Invasive Ductal Breast Carcinoma vs. Normal | 2.632 |  | <0.05 | 2.339 | Radvanyi |
|  | Invasive Lobular Breast Carcinoma vs. Normal | 2.46 |  | <0.05 | 2.099 | Radvanyi |
| HNRNPA2B1 | Invasive Breast Carcinoma vs. Normal | 2.084 |  | <0.0001 | 9.743 | Gluck |
| HNRNPC | NA | NA |  | NA | NA | NA |
| HNRNPD | NA | NA |  | NA |  | NA |
| PCBP1 | Ductal Breast Carcinoma in Situ vs. Normal | 2.945 |  | <0.05 | 2.978 | Radvanyi |
|  | Invasive Ductal Breast Carcinoma vs. Normal | 2.591 |  | <0.05 | 2.38 | Radvanyi |
| HNRNPF | NA | NA |  | NA | NA | NA |
| RBMX | NA | NA |  | NA |  | NA |
| PTBP1 | Mucinous Breast Carcinoma vs. Normal | 2.911 |  | <0.0001 | 9.405 | TCGA |
|  | Invasive Ductal and Lobular Carcinoma vs. Normal | 2.403 |  | <0.001 | 8.217 | TCGA |
| HNRNPK | NA | NA |  | NA | NA | NA |
| HNRNPL | Invasive Lobular Breast Carcinoma vs. Normal | 2.846 |  | <0.001 | 2.972 | Radvanyi |
|  | Invasive Mixed Breast Carcinoma vs. Normal | 2.748 |  | 0.01 | 2.904 | Radvanyi |
|  | Ductal Breast Carcinoma in Situ vs. Normal | 2.291 |  | <0.05 | 2.014 | Radvanyi |
|  | Invasive Ductal Breast Carcinoma vs. Normal | 2.292 |  | <0.05 | 2.395 | Radvanyi |
| HNRNPM | Invasive Lobular Breast Carcinoma vs. Normal | 3.149 |  | <0.05 | 2.161 | Turashvili |
| SYNCRIP | Ductal Breast Carcinoma in Situ Epithelia vs. Normal | 3.146 |  | <0.0001 | 7.525 | Ma Breast 4 |
|  | Invasive Ductal Breast Carcinoma Epithelia vs. Normal | 2.607 |  | <0.001 | 4.793 | Ma Breast 4 |
|  | Ductal Breast Carcinoma vs. Normal | 2.508 |  | <0.0001 | 8.352 | Richardson Breast 2 |
|  | Invasive Ductal Breast Carcinoma vs. Normal | 3.328 |  | 0.01 | 3.604 | Radvanyi |
| HNRNPR | Invasive Mixed Breast Carcinoma vs. Normal | 2.125 |  | <0.05 | 3.021 | Radvanyi |
| HNRNPU | Invasive Ductal Breast Carcinoma vs. Normal | 2.032 |  | <0.05 | 2.221 | Turashvili |
| HNRNPA0 | Mucinous Breast Carcinoma vs. Normal |  | -2.187 | <0.0001 | -5.845 | TCGA |
|  | Invasive Breast Carcinoma Stroma vs. Normal |  | -2.866 | <0.0001 | -15.05 | Finak |
| HNRNPA1 | Invasive Breast Carcinoma Stroma vs. Normal |  | -59.669 | <0.0001 | -24.3 | Finak |
| HNRNPA2B1 | Invasive Breast Carcinoma Stroma vs. Normal |  | -21.08 | <0.0001 | -20.648 | Finak |
| HNRNPC | Invasive Breast Carcinoma Stroma vs. Normal |  | -9.419 | <0.0001 | -22.213 | Finak |
| HNRNPD | Invasive Ductal Breast Carcinoma Stroma vs. Normal |  | -2.697 | <0.001 | -4.373 | Ma Breast 4 |
|  | Invasive Breast Carcinoma Stroma vs. Normal |  | -18.084 | <0.0001 | -21.99 | Finak |
| PCBP1 | Invasive Breast Carcinoma Stroma vs. Normal |  | -23.564 | <0.0001 | -24.644 | Finak |
| HNRNPF | Invasive Breast Carcinoma Stroma vs. Normal |  | -6.675 | <0.0001 | -21.833 | Finak |
|  | Invasive Ductal Breast Carcinoma Stroma vs. Normal |  | -2.24 | <0.001 | -4.479 | Karnoub |
| RBMX | Invasive Breast Carcinoma Stroma vs. Normal |  | -4.765 | <0.0001 | -18.448 | Finak |
| PTBP1 | Invasive Ductal Breast Carcinoma Stroma vs. Normal |  | -7.915 | 0.001 | -3.632 | Karnoub |
| HNRNPK | Invasive Breast Carcinoma Stroma vs. Normal |  | -33.801 | 2.13E-32 | -24.802 | Finak |
| HNRNPL | NA |  | NA | NA | NA | NA |
| HNRNPM | NA |  | NA | NA |  | NA |
| SYNCRIP | Invasive Ductal Breast Carcinoma Stroma vs. Normal |  | -2.246 | <0.001 | -4.374 | Ma Breast 4 |
| HNRNPR | Ductal Breast Carcinoma in Situ vs. Normal |  | -3.387 | <0.05 | -2.858 | Radvanyi |
|  | Invasive Ductal Breast Carcinoma vs. Normal |  | -3.04 | <0.01 | -4.635 | Radvanyi |
|  | Invasive Ductal Breast Carcinoma vs. Normal |  | -2.094 | <0.0001 | -11.755 | TCGA |
| HNRNPU | Invasive Lobular Breast Carcinoma vs. Normal |  | -2.55 | <0.0001 | -21.264 | Curtis |
|  | Invasive Ductal Breast Carcinoma vs. Normal |  | -2.722 | <0.0001 | -34.761 | Curtis |
|  | Mucinous Breast Carcinoma vs. Normal |  | -3.037 | <0.0001 | -19.44 | Curtis |
|  | Tubular Breast Carcinoma vs. Normal |  | -3.121 | <0.0001 | -20.788 | Curtis |
|  | Invasive Ductal and Invasive Lobular Breast Carcinoma vs. Normal |  | -2.664 | <0.0001 | -19.526 | Curtis |
|  | Ductal Breast Carcinoma in Situ vs. Normal |  | -3.048 | <0.0001 | -10.177 | Curtis |
|  | Breast Carcinoma vs. Normal |  | -2.812 | <0.0001 | -9.178 | Curtis |
|  | Invasive Breast Carcinoma vs. Normal |  | -2.094 | <0.0001 | -7.475 | Curtis |
|  | Medullary Breast Carcinoma vs. Normal |  | -2.665 | <0.0001 | -10.208 | Curtis |
|  | Benign Breast Neoplasm vs. Normal |  | -3.082 | <0.05 | -4.891 | Curtis |
|  | Invasive Breast Carcinoma Stroma vs. Normal |  | -10.654 | <0.0001 | -15.338 | Finak |
