# Supplementary material for: Identification of therapeutic targets and prognostic biomarkers from the hnRNP family in invasive breast carcinoma
Source: Aging (Albany NY). 2021 Jan 20;13(3):4503–21. doi: 10.18632/aging.202411 (PMC7906176; doi:10.18632/aging.202411)
Supplement: Supplementary Table 2 [file aging-13-202411-s003.pdf]

Supplementary Table 2. Correlation of hnRNPs mRNA expression and clinical prognosis in BRCA with LNM by kaplan-meier plotter.

Gene	OS				RFS			
	N		HR	Log rank *P*	N		HR	Log rank *P*
	Low	High			Low	High		
HNRNPA0	106	207	0.65(0.44-0.97)	0.033	834	299	0.58(0.45-0.75)	2.00E-05
HNRNPA2B1	127	50	0.72(0.40-1.29)	0.26	358	366	0.72(0.56-0.92)	0.0091
HNRNPC	55	122	0.34(0.20-0.58)	3.60E-05	187	537	0.52(0.40-0.68)	8.60E-07
HNRNPD	135	178	0.78(0.53-1.16)	0.22	391	742	0.81(0.66-0.99)	0.039
PCBP1	80	233	0.71(0.47-1.08)	0.11	351	782	0.90(0.73-1.11)	0.31
HNRNPF	80	233	0.65(0.43-0.98)	0.038	282	851	0.80(0.64-0.99)	0.041
PTBP1	81	232	0.66(0.43-1.01)	0.056	791	342	1.25(1.02-1.54)	0.032
HNRNPK	78	235	0.57(0.38-0.85)	0.0054	771	362	0.71(0.57-0.89)	0.0027
HNRNPL	223	90	1.37(0.92-2.05)	0.12	295	838	1.73(1.33-2.23)	2.50E-05
HNRNPM	52	125	1.62(0.79-3.31)	0.19	536	188	1.42(1.08-1.86)	0.011
SYNCRIP	159	154	1.32(0.89-1.96)	0.16	702	431	1.84(1.50-2.24)	1.20E-09
HNRNPR	83	94	1.55(0.89-2.70)	0.12	497	227	0.59(0.44-0.80)	0.00056
HNRNPU	59	118	2.11(1.06-4.19)	0.029	180	544	0.71(0.54-0.94)	0.015
